# Stepping It Up: Walking Behaviors in Children Transitioning from 5th to 7th Grade

**DOI:** 10.3390/ijerph15020262

**Published:** 2018-02-03

**Authors:** Sharon E. Taverno Ross, Morgan N. Clennin, Marsha Dowda, Natalie Colabianchi, Russell R. Pate

**Affiliations:** 1 Department of Health and Physical Activity, University of Pittsburgh, 32 Oak Hill Court, Pittsburgh, PA 15261, USA; 2 Department of Exercise Science, University of South Carolina, 921 Assembly Street, Columbia, SC 29208, USA; clennin@email.sc.edu (M.N.C.); mdowda@mailbox.sc.edu (M.D.); rpate@mailbox.sc.edu (R.R.P.); 3 Department of Health and Fitness, University of Michigan, 1402 Washington Heights, Ann Arbor, MI 48109, USA; colabian@umich.edu

**Keywords:** school-aged, physical activity, walking, transportation, walkability, Walk Score^®^, child

## Abstract

The purpose of this study was to (1) describe children’s walking behaviors in 5th to 7th grade and change over time and (2) examine associations between walking behaviors and Walk Score^®^. Participants consisted of *n* = 586 students from the Transitions and Activity Changes in Kids (TRACK) Study. Children reported any walking behavior (e.g., exercise and transportation) over the past five days. Walk Score was calculated based on children’s home address. Descriptive statistics summarized walking behaviors by gender and time, and repeated measure mixed models examined the relationship between walking behaviors and Walk Score. Approximately 46.8% and 19.2% of 5th grade children reported walking for exercise and transportation, respectively, and these percentages declined through 7th grade. Girls reported higher levels of total walking behavior and walking for exercise than boys (*p* < 0.001). Girls with a higher Walk Score had 63% higher odds of reporting walking for transportation than girls with a lower Walk Score (OR = 1.63, 95% CI = 1.02, 2.62). Walking behaviors among children were infrequent with significant declines over time, and of the nine associations examined with Walk Score, only one was significant. Efforts should prioritize frequent walking behavior and community design to increase children’s physical activity.

## 1. Introduction

In 2015, the Surgeon General released “Step It Up! The Surgeon General’s Call to Action to Promote Walking and Walkable Communities” [1] to promote physical activity across all ages and abilities in the U.S. Walking at a moderate pace has been associated with health benefits for both children and adults, including reducing the risk of chronic disease [2]. Engaging in physical activity throughout the day, including brisk walking, is important to help children meet the recommended guidelines of 60 min or more of moderate-to-vigorous intensity physical activity per day [1,3]; however, very few U.S. children are achieving this recommendation [4]. Further, there is a marked decline in physical activity as children transition from childhood into adolescence [4,5,6]. 

Walkability can be defined as “the extent to which characteristics of the built environment and land use may or may not be conducive to residents in the area walking for either leisure, exercise or recreation, to access services, or to travel to work” [7]. Aspects of the built environment, including neighborhood walkability, have been linked to physical activity in children and youth. According to a 2011 review of the neighborhood environment and physical activity among youth, the most consistent associations were among objectively measured neighborhood environmental attributes and (self- or parent-) reported physical activity [8]. Specifically, the most supported built environment correlates for children’s physical activity levels were walkability, traffic speed/volume, access/proximity to recreation facilities, land-use mix, and residential density. 

Surprisingly little is known about the walking behaviors of children, particularly during this developmental transition. There is evidence that children are more likely to walk or cycle to school if they live closer to school compared with those who live further away [9,10]. One study by Chillón et al. (2015) characterized the distance thresholds of active commuting to school as changing from 1.4 km at 10 years, to 1.6 km at 11 years, and to 3 km at 14 years [11]. To our knowledge, few studies have examined or described in detail the walking behaviors of children during the transition from elementary school to middle school (5th to 7th grade) and the relationship with walkability of the neighborhoods within which they live. The purpose of this study is to examine walking behaviors and neighborhood walkability in children from 5th to 7th grade. This study has two aims: (1) to describe children’s walking behaviors and change in those behaviors over time (by gender); and (2) to examine the association between an objective measure of neighborhood walkability (Walk Score^®^) and children’s walking behaviors (by gender).

## 2. Materials and Methods

### 2.1. Participants

Data were taken from the Transitions and Activity Changes in Kids (TRACK) Study, a multi-level, longitudinal study of influences on changes in children’s physical activity as they transitioned from elementary (2010) to middle school (2012). The original sample of participants in the 5th grade was *n* = 1083. Fifty-four percent of the sample was female with an average age of 10.5 (0.6) years. Approximately 38% of the sample was white, 34.1% black, and 10.8% Hispanic, and 17.6% were of another race/ethnicity. 

Participants were recruited from two school districts (Site A and Site B) in mid-sized cities in South Carolina. Both counties within which the districts were located can be defined as “urbanized areas” according to criteria for the 2010 Census [12]. The TRACK study included participants from 21 elementary schools (Kindergarten-5th) with an average enrollment of 477, and 13 middle schools (6th–8th grade) with an average enrollment of 656 students. As such, participants changed schools from elementary to middle school during the course of the study. 

Research staff met with district superintendents and administrators prior to soliciting school participation. Recruitment assemblies were held in all schools, during which 5th graders were invited to participate in the study and received information regarding the data collection procedures. Sixty-four percent of students at Site A and 57% of students at Site B provided consent and assented to participating in the study. For the 5th grade students, informed consent packets were sent home from school for the children’s parents to read, complete, and return. Children also gave their written assent before any study procedures began. Consent and written assent was given in Grade 5 for all three years of data collection; however, children provided verbal assent in Grades 6 and 7 to continue their participation. The Institutional Review Board at the University of South Carolina approved all study protocols (Pro00003730; approval date 12 August 2009).

Students were excluded from the study only if they had (a) an orthopedic or other condition that would invalidate the measure of physical activity (e.g., wheelchair-bound) and/or (b) intellectual limitations that prevented participants from completing the surveys. Consenting students were representative of age, gender, and race/ethnicity of the students attending schools in those districts.

For the current sample, we included children in both the 5th and 7th grade who had complete data on the variables of interest. A total of 764 students reported walking behavior in the 5th grade (baseline); however, 178 students were not included due to missing data on walking behavior in 7th grade (*n* = 136) or demographic variables of interest (*n* = 42). The present analyses included data on a total of 586 students (*n* = 263 boys and *n* = 323 girls) who had complete data in both 5th and 7th grades for the variables of interest. With the exception of race, there were no statistically significant demographic differences between the two samples.

### 2.2. Data Collection

In Grades 5, 6, and 7, data collection procedures occurred over two sessions at the school with each participant and were administered by a trained measurement team. While the participants completed a battery of research procedures, the data for the current study was collected through a survey instrument administered to participants in Grades 5, 6, and 7 during the second data collection session.

### 2.3. Measures

#### 2.3.1. Child Walking Behavior

The Physical Activity Choices (PAC) instrument was used to measure participation in walking behaviors in Grades 5, 6, and 7. The PAC was based on the 3DPAR instrument [13], but instead of asking about the activity performed during specific time blocks, participants responded about any participation in the activity over the past five days (which may or may not have included any weekend days). Participants completed the PAC using a computer-assisted, self-administered protocol and were guided through a list of 56 activities (7 sedentary, 49 physical activities). If they responded “yes,” they were asked about the number of days the activity occurred and how long they did the activity on each occasion (average minutes). Assessing physical activity over multiple days, as done here, has been shown to provide valid and reliable estimates of usual activity in children as young as 5th grade [14,15]. For the present analysis, child responses for items regarding “any walking for exercise,” “any walking for transportation,” and total walking behavior (exercise + transportation) were used.

#### 2.3.2. Neighborhood Walkability

Walk Score^®^ was used to assess neighborhood walkability among TRACK participants [16]. Each participant’s home address was manually entered into the Walk Score site by trained research staff. For each address, the Walk Score algorithm produced a score ranging from 0 to 100 based on the ease of walking to various amenities in the neighborhood. For a given address, an algorithm is used to generate a Walk Score ranking based on distance to various types of amenities (e.g., education, retail stores, food/restaurants, recreation/parks, entertainment). The algorithm uses a distance–decay function to generate a score for each type of amenity included in the Walk Score measure. For instance, amenities located closer to a child’s home received a higher score than amenities located further away. Scores are summed across each amenity type and normalized to produce the final measure [17,18,19,20]. 

For the present study, the categorical expression of the Walk Score variable was informed by the established Walk Score cutpoints [21]: 0–24: “car-dependent (almost all errands require a car)”; 25–49: “car-dependent (most errands require a car)”; 50–69: “somewhat walkable (some errands can be accomplished on foot)”; 70–89: “very walkable (most errands can be accomplished on foot)”; 90–100: “walker’s paradise (daily errands do not require a car).” Due to the small sample sizes within the High and Moderate Walk Score cutpoints, the Walk Score variable was dichotomized for the current analyses into Very Low Walkability (Walk Score ranking <25) and Low/Moderate Walkability (Walk Score ranking ≥25). 

#### 2.3.3. Sociodemographics

Participants reported their age, gender, and race/ethnicity. For race, they were asked to check as many categories as applied (white, black/African American, Asian, American Indian/Alaskan Native, and other). They were also asked to identify whether they considered themselves Hispanic or Latino. Race/ethnicity responses were re-coded as black, white, Hispanic, and other/mixed race. Parents reported their highest level of education and item responses were re-coded to “high school or less” and “more than high school.”

### 2.4. Statistical Analyses

Descriptive statistics were calculated for the sociodemographic characteristics of the total sample and by gender. *t*-Tests and chi-square analyses were used to determine if there were differences in the characteristics by gender. Unadjusted frequencies for walking behaviors (i.e., walking for exercise and walking for transportation) were calculated for the total sample and by gender in Grades 5, 6, and 7. Chi-square analyses were used to determine if there were differences in the characteristics by gender or changes in walking behavior across grade level.

Repeated mixed model regression analysis (Proc Glimmix, with binomial distribution) was used to determine whether there were differences by grade level or time for walking behaviors (walk for exercise, walk for transportation, or total walking behavior). Models were also run separately for boys and girls. Models were adjusted for race/ethnicity, parent education, and gender for total group, with school as a random effect. 

Mixed model regression (Proc Glimmix with binomial or multinomial distribution) was also used to determine differences in child walking behaviors by neighborhood walkability (i.e., categorical Walk Score ranking). Models were performed for the total sample and separately by gender and were calculated adjusting for age, race/ethnicity, and parent education. School nested within location (i.e., study site) was treated as a random effect. All analyses were conducted with SAS 9.3 (SAS Institute Inc., Cary, NC, USA), and statistical significance was set at *p* < 0.05.

## 3. Results

### 3.1. Sociodemographic Characteristics

Descriptive statistics for the sociodemographic characteristics of the total sample and by gender are presented in Table 1. The mean age of the total sample was 10.5 years (SD = 0.54). Approximately 40.3% of the children were categorized as black, 36.4% were white, 8.5% were Hispanic, and 14.7% were of a different race/ethnicity. The mean ± SD Walk Score ranking for the total group was 13.3 (17.1). Approximately 80.2% and 74.3% of boys and girls, respectively, were categorized as living in an area with a Very Low Walk Score ranking. There were no statistically significant differences by gender for any of the sociodemographic or walking environment variables.

### 3.2. Walking Behaviors

Table 2 includes reported walking behaviors in the past five days (% Yes) for the total group and boys and girls by grade-level. At baseline, nearly 55% of the sample reported walking for exercise and/or walking for transportation in the past five days. Descriptively, at each grade level, a higher proportion of children reported walking for exercise than walking for transportation in the past five days. For example, approximately 46.8% and 19.2% of 5th grade children reported walking for exercise and walking for transportation, respectively. In the total group, there was a marked decline in both walking for exercise (*p* < 0.001) and walking for transportation (*p* = 0.04) from Grade 5 to Grade 7. 

Overall, girls reported significantly more walking for exercise and total walking behavior than boys (*p* < 0.001). When looking at differences across time for boys and girls, there were significant declines in total walking behavior for both genders (*p* < 0.001). There was also a significant decline in walking for exercise in both boys and girls from 5th to 7th grade. Specifically, nearly 55% of girls and 38% of boys reported walking for exercise in Grade 5, and this declined to 26.9% of girls and 17.1% of boys in the 7th grade. Descriptively, we saw a similar pattern in walking for transportation for both genders, but this decline did not reach statistical significance. 

### 3.3. Association between Walking Behaviors and Neighborhood Walkability

Table 3 presents the odds ratios (ORs) and 95% confidence intervals (CIs) for walking for exercise, walking for transportation, and total walking behavior across Walk Score categories for the total group and by gender. After adjusting for covariates, the odds of reporting any type of walking behavior did not vary by Walk Score category over time. Next, non-significant interactions between Walk Score and time were dropped. Models were then rerun to examine the relationship between each type of walking behavior, neighborhood Walk Score, and time. Over time, the odds of reporting walking for exercise and total walking behavior decreased among girl, boys, and the total group. No significant change in student-reported walking for transportation was observed. With respect to neighborhood walkability, Walk Score was significantly associated with walking for transportation among girls. Specifically, the odds of reporting walking for transportation were 63% higher among girls residing in neighborhoods with a Low/Moderate Walk Score compared to girls residing in a neighborhood with a Very Low Walk Score (OR = 1.63, 95% CI = 1.02, 2.62). Walk Score ranking was not significantly associated with walking for exercise or total walking behavior among girls, and was not associated with any type of walking behavior among the total group or boys. 

## 4. Discussion

Data from the current sample suggests that, overall, the percentage of children walking for exercise and walking for transportation is infrequent; furthermore, levels decline markedly during the transition from elementary to middle school. The Surgeon General released “Step It Up! The Surgeon General’s Call to Action to Promote Walking and Walkable Communities” with the goal of promoting physical activity across all age sectors and abilities in the U.S. [1]. Engaging in walking and wheelchair rolling to achieve daily recommended physical activity levels can help to prevent and reduce the risk of chronic disease, premature death, as well as support positive mental health and healthy aging. However, findings from the present study suggest that there is still much work that can be done to promote walking and walkable communities for these children. 

There were differences in terms of the type of walking behavior in which children engaged. Descriptively, the percentage of children reporting walking for exercise in the past five days was higher in both boys and girls compared to walking for transportation. This is likely due to the low walkability levels of their communities, including having limited destinations to walk to (e.g., living in rural or suburban areas, schools located far from home). However, this also suggests that achieving moderate levels (>50% of girls in the 5th grade) of walking for exercise is feasible even in neighborhoods with low walkability. We know from the literature that physical activity is associated with positive health outcomes in both children and adults [2]. As such, innovative and multisector strategies that engage children, parents, schools, community organizations, and local government officials, should be promoted in order to better support walking behaviors over time during the transition from elementary to middle school. 

Overall, girls have lower levels of physical activity and are less likely to meet physical activity guidelines when compared with boys [4,5,6,22]. However, in the current study, girls reported higher levels of total walking behaviors in 5th, 6th, and 7th grade than boys. This corroborates with a previous study of high school students, where walking was the most commonly reported physical activity engaged in over a period of 12 months, and girls reported higher levels of walking than boys [23]. In the present study, the decline in total walking for girls was drastic and troubling (~64% in 5th grade vs. ~35% in 7th grade). It is possible that walking behavior is a type of physical activity that should be promoted to help girls meet physical activity guidelines. This may be especially important and necessary during the transition from elementary to middle school, as demonstrated by the marked decline in walking behaviors for girls in current data. The results of the regression models found that the odds of reporting walking for transportation were 63% higher among girls residing in neighborhoods with a Low/Moderate Walk Score compared to girls residing in a neighborhood with a Very Low Walk Score. This suggests that improving neighborhood environments to be more conducive to walking, particularly having amenities located closer to where children live, may be beneficial to walking behaviors, particularly for young girls. 

Overwhelmingly, participants had low neighborhood walkability, and the majority of children (80.2% and 74.3% of boys and girls, respectively) were categorized as living in a car-dependent area with a Very Low Walk Score. In the two sites (communities) from which these participants were recruited, the traditional categorization of Walk Score was not successful. Specifically, this study included participants from 21 elementary schools (K-5) and 13 middle schools (6–8) in two school districts in urbanized areas in South Carolina. While both sites are characterized as mid-sized cities (40,000–67,000 residents), they differ from traditional metropolitan cities that have been studied in previous literature [24,25,26,27,28]. The Walk Score variable has been previously associated with minutes of walking for transportation and walking for exercise in urban adults [28] as well as with moderate-to-vigorous physical activity in seniors, adults, and teenagers, but not children, living in three urban areas [29]. In the current study, Walk Score ranking was generally not associated with child-reported walking for exercise or walking for transportation. It is possible that an objective measure of total amount of walking, such as step counts or distance traveled, may have allowed us to see significant associations between Walk Score and walking behavior. Additionally, using the standard Walk Score cutpoints resulted in very low variability within our sample, and likely contributed to the null results. That is, the tool may not have captured other important factors that could influence the walking behaviors of children living in “mid-sized cities.”

To our knowledge, this is one of the first studies to characterize longitudinal walking behaviors for children during the transition from elementary to middle school. Another strength of the study is the inclusion of a large, diverse sample of both boys and girls (nearly 64% non-white). However, the study is not without limitations. The measure of walking behavior was based on child-report of activities engaged in during the past five days (yes/no) and may not be representative of their typical walking behaviors. Further, the measure of neighborhood walkability, Walk Score^®^, resulted in low variability, which may have influenced the findings. 

## 5. Conclusions

This study found that walking behaviors among children were infrequent, with significant declines over time for both boys and girls during the transition from elementary to middle school. Interestingly, walkability, as measured by the Walk Score, was not generally associated with children’s walking behaviors in this sample from urbanized areas in South Carolina. Based on the results of the National Physical Activity Plan Alliance’s national assessment of walking and walkability in the United States [30], and in line with the results of this study, there is much room for improvement on a community level in meeting selected standards for participating in walking and providing physical and social supports for walking behavior. To maintain walking behavior over time, a multi-pronged approach engaging a variety of stakeholders, and targeting both walking behavior and community design, should be prioritized.

## Figures and Tables

**Table 1:** Sociodemographic characteristics of 5th grade participants in the Transitions and Activity Changes in Kids (TRACK) Study (*n* = 586).

Child Characteristic	Total *n* = 586	Boys *n* = 263	Girls *n* = 323
Age, years (Mean, SD)	10.5 (0.54)	10.5 (0.55)	10.5 (0.53)
Race/ethnicity (%)			
Black	40.3%	42.2%	38.8%
Hispanic	8.5%	9. 5%	7.8%
White	36.4%	33.5%	38.8%
Other/mixed race	14.7%	14.8%	14.6%
Parent Education (%)			
High school or less	42.5%	41.1%	43.3%
Walking Environment	*n* = 572	*n* = 257	*n* = 315
Walk Score^®^ (Mean, SD)	13.3 (17.1)	11.8 (16.5)	14.6 (17.5)
Very Low Walkability (Walk Score ranking < 25) (%)	76.4%	79.8%	73.7%
Low/Moderate Walkability (Walk Score ranking ≥ 25) (%)	23.6%	20.2%	26.4%

NOTE: Walk Score variable had fewer participants with this data (*n* = 572); there were no statistically significant differences in sociodemographic characteristics or walking environment between boys and girls.

**Table 2:** Walking behaviors (% Yes) in the past five days by gender and grade-level among participants in the TRACK study (*n* = 586).

Walking Behavior	Total (*n* = 586)				Boys (*n* = 263)				Girls (*n* = 323)			
	5th	6th	7th	*p*-Value	5th	6th	7th	*p*-Value	5th	6th	7th	*p*-Value
Walk for Exercise ***	46.8	30.2	22.4	<0.001	37.6	22.6	17.1	<0.001	54.5	35.9	26.9	<0.001
Walk for Transportation	19.2	15.5	13.9	0.04	18.3	16.5	13.3	0.29	20.1	14.6	14.2	0.09
Total Walk (Exercise + Transportation) ***	54.9	39.0	31.6	0.53	46.4	31.8	27.4	<0.001	61.9	44.7	35.3	<0.001

NOTE: Differences between boys and girls *** *p* < 0.001; Gender by time interactions non-significant for walking behaviors.

**Table 3:** Repeated measure mixed model logistic regression—relationship between Walk Score^®^ ranking and walking for exercise, walking for transportation, and total walking behavior.

Type of Walking Behavior	Repeated Measure Logistic Regression *p*-Values			Walk Score Ranking
	Walk Score	Time	Walk Score * Time	OR (95% CI)
TOTAL GROUP (*n* = 572)				
Walk for exercise	0.34	<0.001	NS	0.86 (0.63, 1.18)
Walk for Transportation	0.32	0.06	NS	1.19 (0.85, 1.66)
Total Walk (Exercise + Transportation)	0.64	<0.001	NS	1.07 (0.81, 1.41)
BOYS (*n* = 257)				
Walk for exercise	0.50	<0.001	NS	0.84 (0.52, 1.39)
Walk for Transportation	0.3331	0.3529	NS	0.79 (0.49, 1.27)
Total Walk (Exercise + Transportation)	0.59	<0.001	NS	0.88 (0.56, 1.39)
GIRLS (*n* = 315)				
Walk for exercise	0.5655	<0.001	NS	0.90 (0.62, 1.31)
Walk for Transportation	0.04	0.13	NS	1.63 (1.02, 2.62)
Total Walk (Exercise + Transportation)	0.29	<0.001	NS	1.20 (0.85, 1.73)

NOTE: OR: odds ratio; CI: confidence interval; NS: non-significant *p*-value; Final sample was *n* = 572 due to fewer participants with this data; Analyses adjusted for gender, race, and parent education; Models with non-significant interactions were rerun after dropping interaction term; Walk Score ranking treated as dichotomous variable (Very Low = Walk Score ranking <25; Low/Moderate = Walk Score ranking ≥ 25).
